# Phylogeography indicates incomplete genetic divergence among phenotypically differentiated montane forest populations of *Atlapetesalbinucha* (Aves, Passerellidae)

**DOI:** 10.3897/zookeys.809.28743

**Published:** 2018-12-19

**Authors:** Alberto Rocha-Méndez, Luis A. Sánchez-González, Enrique Arbeláez-Cortés, Adolfo G. Navarro-Sigüenza

**Affiliations:** 1 Museo de Zoología, Facultad de Ciencias, Universidad Nacional Autónoma de México, Apartado Postal 70-399, México City 04510, México Universidad Nacional Autónoma de México México City Mexico; 2 Grupo de Estudios en Biodiversidad, Escuela de Biología, Facultad de Ciencias, Universidad Industrial de Santander, Carrera 27 Calle 9. Bucaramanga, Santander, Colombia Universidad Industrial de Santander Bucaramanga Colombia

**Keywords:** allotypy, coalescent, Last Glacial Maximum, mtDNA, montane forest, Pleistocene, phylogeography, plumage differentiation

## Abstract

The White-naped Brushfinch (*Atlapetesalbinucha*) comprises up to eight allopatric subspecies mainly identified by the color of the underparts (gray vs. yellow belly). Yellow and gray bellied forms were long considered two different species (*A.albinucha* and *A.gutturalis*), but they are presently considered as one polytypic species. Previous studies in the genus *Atlapetes* have shown that the phylogeny, based on molecular data, is not congruent with characters such as coloration, ecology, or distributional patterns. The phylogeography of *A.albinucha* was analyzed using two mitochondrial DNA regions from samples including 24 different localities throughout montane areas from eastern Mexico to Colombia. Phylogeographic analyses using Bayesian inference, maximum likelihood and haplotype network revealed incomplete geographic structure. The genetic diversity pattern is congruent with a recent process of expansion, which is also supported by Ecological Niche Models (ENM) constructed for the species and projected into three past scenarios. Overall, the results revealed an incomplete genetic divergence among populations of *A.albinucha* in spite of the species’ ample range, which contrasts with previous results of phylogeographic patterns in other Neotropical montane forest bird species, suggesting idiosyncratic evolutionary histories for different taxa throughout the region.

## Introduction

Phylogeographic analyses of widespread Neotropical montane forest bird species have indicated different levels of geographic structure in the variation of mitochondrial DNA (mtDNA) among populations (Bonaccorso et al. 2008, Navarro-Sigüenza et al. 2008, Barrera-Guzmán et al. 2012, Ortíz-Ramírez et al. 2016). Deep genetic divergence may suggest a long history of geographic isolation, usually accompanied by phenotypic divergence, whereas a shallow genetic differentiation has been attributed to possible scenarios of either recent range expansions or recent divergence (Cortés-Rodríguez et al. 2008, Arbeláez-Cortés et al. 2010).

The genus *Atlapetes* comprises a group of Neotropical finches inhabiting mainly humid montane forests from Mexico to northern Argentina (Paynter 1972, 1978), and has been considered one of the most species-rich clades (nearly 30 recognized species) among the New World passerines (Paynter 1972, 1978, Sánchez-González et al. 2015). Diversification in this group started about 5.2–3.2 Mya, and has probably occurred mainly due to changes in the range of montane forests related to the Pleistocene glacial cycles (Ricklefs and Latham 1992, Hooghiemstra et al. 2006, Sánchez-González et al. 2015). *Atlapetes* also provides a complex and intriguing case of evolutionary differentiation in plumage color (Paynter 1972, 1978, Remsen and Graves 1995). Most *Atlapetes* species have either yellow or gray underparts, which has led to the distinction of two main plumage patterns distributed in a “leapfrog” fashion along the Andes (Remsen 1984). Paynter (1972, 1978) included yellow and gray taxa in two different phylogenetic groups; a third group included yellow-plumaged species with bicolored crowns (*A.albinucha*, *A.gutturalis*, and *A.pallidinucha*). This phenotypically-based arrangement suggested that similarly plumaged species may have shared a common ancestor. However, Remsen and Graves (1995) proposed that some yellow and gray taxa may be representatives of the same species, suggesting that plumage differentiation patterns may be adaptive, as species with pale gray colors are generally found in drier and higher elevation habitats, whereas yellow-colored taxa are generally found in humid and lower elevations (Remsen and Graves 1995, García-Moreno and Fjeldså 1999).

**Figure 1.**
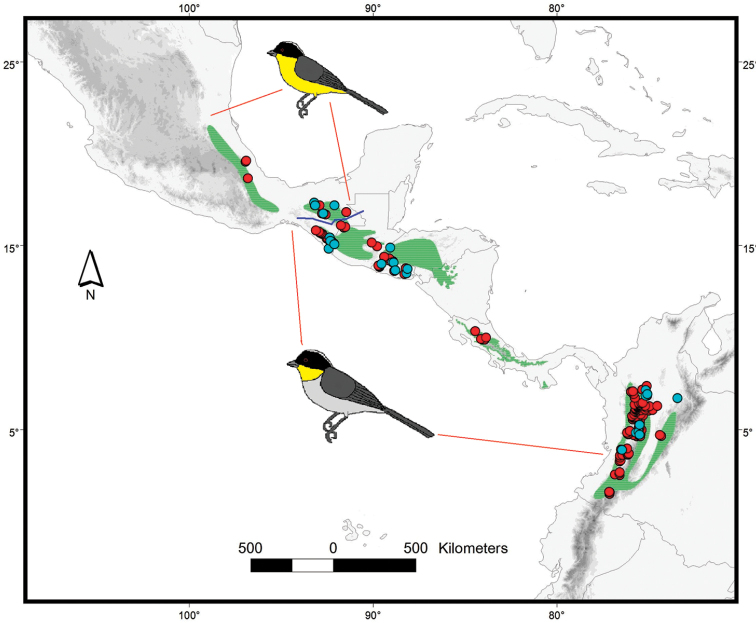
*Atlapetesalbinucha* distribution shown in green stapled lines, based on Sánchez-González et al. (2015) and Natureserve (http://natureserve.org). Blue dots depict tissue samples used in the present study. Red dots depict records of the species used to construct the distribution model. Bird pictures depict the geographic regions where color morphs are found. Blue line depicts the location of the putative distribution barrier of the morphs in Chiapas.

*Atlapetesalbinucha* (White-naped Brushfinch) is a widely distributed species found in montane regions from eastern Mexico to Colombia (AOU 1998, del Hoyo et al. 2011, Gill and Donsker 2015, Remsen et al. 2015). This species inhabits mainly humid and temperate montane forests (900 to 3000 m), as well as upper tropical zones and edges of clearings of cloud forests (Dwight and Griscom 1921, Paynter 1978, Howell and Webb 1995, del Hoyo et al. 2011). Up to eight allopatric subspecies showing two well-differentiated plumage coloration patterns with clear geographic structure have been recognized (Figure 1, del Hoyo et al. 2011, Remsen et al. 2015, Gill and Donsker 2018). Phenotypic differentiation in this taxon led some researchers to consider such differentiated populations as two species (Dwight and Griscom 1921, Howell and Webb 1995, Navarro-Sigüenza and Peterson 2004) or as part of a polytypic species (Paynter 1978, Remsen and Graves 1995, AOU 1998, del Hoyo et al. 2011, Gill and Donsker 2015, Remsen et al. 2015). Differences between subspecies with pale-gray underparts are apparently largely clinal, and are based in subtle variations of size and color (Paynter 1978); while color morphs with yellow- and pale-gray underparts are allopatric (Figure 1), and their ranges are separated by the low valley of Río Grijalva (1000 m) in Chiapas (southeastern Mexico), where the two morphotypes are less than 100 km apart (Paynter 1978).

Paynter (1972, 1978) reviewed extensively the taxonomy and geographic variation in *A.albinucha* complex and suggested that plumage differentiation in these nearly parapatric populations were not indicative of a high genetic differentiation nor the product of ecological exclusion, but the result of a low river barrier, therefore implying isolation due to environmental factors as the main cause for this phenotypic differentiation. Current taxonomic schemes have adopted the proposal that *A.albinucha* represents a single polytypic species (Paynter 1978, Remsen and Graves 1995, AOU 1998, del Hoyo et al. 2011, Gill and Donsker 2015, 2018, Remsen et al. 2015). A study using mtDNA genetic analyses (Sánchez-González et al. 2015) at the genus level showed a phylogenetic reconstruction in which both yellow- and gray-plumaged birds in the *A.albinucha* complex were recovered mixed in a monophyletic group, sister to *A.pileatus* and well separated from the rest of *Atlapetes*, partially supporting the conclusions of Paynter (1978).

Here, using an extensive sampling for *A.albinucha* (39 individuals from 24 localities in four countries), we tested: a) if yellow- and gray-plumaged groups are reciprocally monophyletic, b) if there is phylogeographic structure in this widespread taxon across their range, and c) if past reconstructions of the environmental conditions were *A.albinucha* ranges at present indicate distributional changes that may be related with their genetic-geographic variation. These questions were approached using a mtDNA assessment of populations included within this complex, as well as environmental niche modelling analyses based on records of voucher specimens in biological collections.

## Materials and methods

### Taxon sampling

Tissue and blood samples of *A.albinucha* were obtained from different museum collections in Mexico, USA, and Colombia, spanning the whole distribution of the species (Table 1, see Acknowledgements), except from two subspecies endemic to Panama (*brunnescens* and *azuerensis*). We also supplemented our study with two published sequences of *Atlapetespileatus*, and two from *Arremonbrunneinucha* to be used as outgroups (Klicka et al. 2007, DaCosta et al. 2009). Overall, we analyzed 39 samples from *A.albinucha* representing 24 localities from four countries, and four subspecies (Figure 1, Table 1).

**Table 1.** Tissue and blood samples used in this study. Samples of *A.albinucha* species were grouped in five geographic groups: Northern Chiapas (NC, n = 11), Southern Chiapas (SC, n = 11), El Salvador (Sal, n = 7), Honduras (Hon, n = 1), and Colombia (Col, n = 9). One sample of *Atlapetespileatus* tissue was also obtained and added to the analysis.

Sample source	Catalog number	Voucher specimen	State/ Department	Locality	Latitude	Longitude	GenBank Accession Number		Geographic Group
							ND2	Cyt b	
Museo de Zoología "Alfonso L. Herrera", Facultad de Ciencias, UNAM	BONA 33	BONA 33	Chiapas	Carretera estatal Coapilla-Ocotepec km 29 a 5.4 km N de Coapilla	17.31388, -93.2		MH938475	MH938514	NC
	BONA 39	BONA 39	Chiapas	Volcán Tacaná ladera, vereda a Tapalapa, Rancho Chiquihuite	15.0666, -92.08333		MH938476	MH938515	SC
	BONA 52	BONA 52	Chiapas	Volcán Tacaná ladera, vereda a Tapalapa, Rancho Chiquihuite	15.0666, -92.08333		MH938474	MH938513	SC
	BONA 89	BONA 89	Chiapas	Volcán Tacaná ladera, vereda a Tapalapa, Rancho Chiquihuite	15.0666, -92.08333		MH938479	–	SC
	BONA 94	BONA 94	Chiapas	Volcán Tacaná ladera, vereda a Tapalapa, Rancho Chiquihuite	15.0666, -92.08333		MH938477	MH938516	SC
	BMM 577	BMM 577	Chiapas	6 km NE de Pueblo Nuevo; camino a Aurora-Ermita	17.18333, -92.08333		MH938478	MH938517	NC
	BMM 582	BMM 582	Chiapas	6 km NE de Pueblo Nuevo; camino a Aurora-Ermita	17.18333, -92.08333		MH938464	MH938503	NC
	BMM 834	BMM 834	Chiapas	Volcán Tacaná ladera, vereda a Tapalapa, Rancho Chiquihuite	15.0666, -92.08333		KM360517	MH938498	SC
	MOL 13001	MOL 13001	Chiapas	San Nicolás Buenavista, Cerro Huitepec	16.73805, -92.68805		MH938468	MH938507	NC
	MOL 13061	MOL 13061	Chiapas	San Nicolás Buenavista, Cerro Huitepec	16.73805, -92.68805		MH938467	MH938506	NC
	MOL 13130	MOL 13130	Chiapas	San Nicolás Buenavista, Cerro Huitepec	16.73805, -92.68805		MH938466	MH938505	NC
	SIT 105	SIT 105	Chiapas	CarreteraCopainalá-Ocotepec km 38 a 95.5 km N de Coapilla	17.16891, -93.14533		MH938481	MH938519	NC
	SIT 146	SIT 146	Chiapas	Coapilla a 6.5 km N	17.17413, -93.14636		MH938463	MH938502	NC
	SIT 147	SIT 147	Chiapas	Coapilla a 6.5 km N	17.17413, -93.14636		MH938472	MH938511	NC
	SIT 157	SIT 157	Chiapas	Coapilla a 6.5 km N	17.17413, -93.14636		MH938462	MH938501	NC
	SIT 158	SIT 158	Chiapas	Coapilla a 6.5 km N	17.17413, -93.14636		MH938480	MH938518	NC
	EAGT 806	EAGT 806	Chiapas	Cerro Mozotal, en la cima	15.4294, -92.3411		MH938458	MH938495	SC
	EAGT 817	EAGT 817	Chiapas	Cerro Mozotal, en la cima	15.4294, -92.3411		MH938457	MH938494	SC
	EAGT 844	EAGT 844	Chiapas	Cerro Boqueron, en la cima	15.23541, -92.30463		MH938473	MH938512	SC
	ZRH 407	ZRH 407	Chiapas	Cerro Mozotal, en la cima	15.4294, -92.3411		MH938460	MH938499	SC
Museo de Zoología Facultad de Ciencias “Alfonso L. Herrera” UNAM	130332	130332	Chiapas	Reserva Ecológica el Triunfo	14.81278, -92.40594		MH938456	MH938492	SC
	130345	130345	Chiapas	Reserva Ecológica el Triunfo	14.81278, -92.40594		KM360516	MH938493	SC
	QRO0272	QRO0272	Querétaro	El Pemoche	21.2263, -99.109694		MH938455	MH938491	
	OVMP227	OVMP227	Jalisco	–	–	–	FJ547292	FJ547251	
University of Kansas, Natural History Museum	EAGT 21	KU 4907	San Miguel	San Miguel	13.48138, -88.1775		GU377050	MH938497	Sal
	EAGT 74	KU 5017	Chalatenango	Concepción Quezaltepec	14.08333, -88.95		MH938459	MH938496	Sal
	OK 56	KU 4961	Morazan	Chilanga	13.71666, -88.11666		MH938465	MH938504	Sal
	CMZF 120	KU 6448	San Vicente	Nuevo Tepetitán	13.64527, -88.78416		MH938471	MH938510	Sal
	LR 58	KU 7704	Chalatenango	La Laguna	14.0666, -88.8666		MH938470	MH938509	Sal
	SLA 165	KU 7775	San Vicente	Nuevo Tepetitán	13.64527, -88.78416		MH938461	MH938500	Sal
	MBR 6584	KU 9400	Santa Ana	Metapán	13.98333, -89.5333		MH938469	MH938508	Sal
Instituto de Investigación de Recursos Biológicos “Alexander von Humboldt”	IAvH-CT-01158	IAvH-11694	Pereira	Parque regional Ucumarí Entre Peña Bonita y Peñas Blancas	4.709233, -75.4907		MH938483	MH938521	Col
	IAvH-CT-01726	IAvH-11946	Aranzazu	Vereda El Laurel, Cuenca Alta del Río Hacienda Termopilas	5.230944, -75.48841		MH938484	–	Col
	IAvH-CT-02391	IAvH-12363	Yotoco	Yotoco	3.87975, -76.443		MH938485	MH938522	Col
	IAvH-CT-04519	IAvH-13101	Santa Rosa de Cabal	Vereda La Linda, Parque Municipal de Campoalegre	4.8675, -75.54666		MH938486	MH938523	Col
	IAvH-CT-04835	IAvH-CT-04835	Anorí	Vereda Santa Gertrudis, Finca La Estrella margen derecha de la Quebrada Santa Gertrudis	7.135444, -75.15527		MH938487	–	Col
	IAvH-CT-07844	IAvH-CT-07844	Amalfi	Vereda Cajamarca, Finca Canales Cuenca de la quebrada Cajamarca	6.8235, -75.15527		MH938488	MH938524	Col
	IAvH-CT-09344	ICN 34591	Amalfi	Vereda El Encanto, La Secreta	6.909167, -75.0766		MH938489	MH938525	Col
	IAvH-CT-09695	IAvH-CT-09695	Pereira	P. Ucumarí, La Pastora	4.814278, -75.69455		MH938490	MH938526	Col
	IAvH-CT-18248	ICN 38086	Santander	Serranía de los Yariguies, Carmen de Chucurí	6.68333, -73.4333		MH938482	MH938520	Col
Marjorie Barrick Museum of Natural History	GAV 1374	MBM 6640	Copán	Copán Ruinas, 10 km ENE	14.86667, -89.05		GU377047	DQ459625	Hon
	DAB1706	MBM 4600	Managua	Chocoyero, Volcán Mombacho, 48km SE Managua	11.829, -85.963		EF529823	EF529932	

### Laboratory procedures

Extraction of DNA from tissue samples was carried out in two laboratory facilities in Mexico and Colombia using the DNeasy Blood & Tissue Kit (Qiagen Inc., Valencia, CA) following manufacturer’s protocols. We amplified two mtDNA genes fragments comprising the NADH dehydrogenase subunit 2 (ND2) and Cytochrome b (Cyt b), which have been shown to successfully assess phylogenetic relationships due to its high probability for tracking recent diversification events (Ball and Avise 1992, Avise 2000). We used primers L5215 (Hackett 1996) and H1064 (Drovetsky et al. 2004) for ND2. The Cyt b was amplified using primers L14990 (Kocher et al. 1989) and H15646 (Sorenson et al. 1999). PCR amplification reactions were performed in 12 to 25 µl reaction mix containing 2 µl of each primer, 2 µl (~10 ng) of DNA and 6 µl Readymix Redtaq (Sigma-Aldrich), or 6 µl of Taq polymerase. PCR products were observed in a 1% agarose gel stained with Ethidium Bromide (EtBr) or EZ-Vision. DNA sequencing was performed at the High Throughput Genomics Center of the University of Washington (USA), Macrogen Inc., Korea, and at the *Servicio de Secuenciación y Análisis Molecular Universidad Nacional* (SSiGMol, Colombia). Sequences were edited and aligned by eye using SEQUENCHER 5.4.6 (Gene Codes Corporation, Ann Arbor, MI USA). Mitochondrial origin for all of our sequences was corroborated in BLAST, through the NCBI server (https://blast.ncbi.nlm.nih.gov/Blast.cgi). Afterwards sequences were aligned using CLUSTALX 2.1 (Thompson et al. 1997) and inspected by eye. Newly generated sequences have been deposited in Gen Bank under accession numbers MH938455 to MH938526.

### Phylogeographic analyses

We conducted analyses using an alignment with both mtDNA loci (ND2 and Cyt B) concatenated. Nucleotide substitution model parameters and partition schemes were estimated for each gene in PARTITIONFINDER (Lanfear et al. 2012), using the Bayesian Information Criterion (BIC) for model selection. Resultant partition schemes and model parameters were used for conducting a phylogenetic reconstruction using the Bayesian inference approach (BI) implemented in MR. BAYES 3.2 (Ronquist et al. 2011) using two independent searches running four Markov-Chains Montecarlo (temperature 0.2) for 10^6^ generations sampling every 1000 generations. Convergence across runs was evaluated using two methods: I) the examination of the standard deviation of split frequencies (with acceptance values <0.01); and II) by verification of parameter estimates in TRACER v1.6 (Rambaut et al. 2014), based on acceptable effective sample sizes (ESS values > 200). After checking for convergence, the first 25 % of the generated trees were discarded as burn-in and the remaining 75 % were kept to calculate posterior probabilities. In addition, we also conducted phylogenetic analyses using maximum likelihood (ML) criteria as implemented in RAXMLGUI 1.5b1 (Stamatakis 2006, 2014, Silvestro and Michalak 2012), using the GTRCAT model for nodal support via 1000 bootstrap iterations using the selected partitions. We considered nodes highly supported when bootstrap values were > 70 % (ML) or when posterior probability values were > 90 % (BI).

Divergence times were estimated through calculation of a maximum clade credibility tree (MCCT) using a Yule speciation process (Yule 1925, Gernhard 2008). Calibration of divergence time estimates was based on mutation rates proposed for the ND2 (0.013 subs/site/lineage/My, Arbogast et al. 2006) and Cyt b (0.01 subs/site/lineage/My, Lovette 2004) loci. To test whether our dataset fits to a strict clock model or to a relaxed clock model, we performed selection tests through the stepping-stone method (Xie et al. 2011) as implemented in MrBAYES 3.2 (Ronquist et al. 2011). Given our partitioning model, the mean marginal likelihood of the strict clock (-Ln 5845.98) performed better than the relaxed clock (-Ln 5913.55). Therefore, chains were run under a strict clock with substitution models according to PARTITIONFINDER for 10^6^ generations and discarded the first 25 % as burn-in. Stationarity was analyzed with TRACER v1.6 (Rambaut et al. 2014). Mean heights and 95 % credibility interval values for node estimates were generated in TREEANNOTATOR v1.8.4 (Drummond et al. 2012) with a posterior probability limit of 0.6. Trees were visualized in FIGTREE v1.4.2 (http://tree.bio.ed.ac.uk/software/figtree/).

Finally, to complement the visualization of the relationships among haplotypes, a haplotype network was constructed using NETWORK 4.6.1.1 (Fluxus Engineering, www.fluxus-engineering.com), through a Median-joining method, assigning equal weights to all variable sites and an epsilon parameter with default values (Ɛ = 0). This method estimates evolutionary relationships among sequences when divergences are recent (Crandall and Templeton 1996, Bandelt et al. 1999).

### Population genetic structure and historical demographic analyses

To analyze the molecular information in the framework of population genetics, we clustered individuals of *A.albinucha* into four groups considering subspecific membership as well as geographic proximity and evidence of montane forest continuity (Table 1): (I) Northern Chiapas (NCh, n = 11; subspecies albinucha), (II) Southern Chiapas (SCh, n = 11; subspecies griseipectus), (III) El Salvador (Sal, n = 7; subspecies griseipectus), Honduras (Hon, n = 1; subspecies fuscipygius), and (IV) Colombia (Col, n = 9; subspecies gutturalis). For each group, genetic diversity was assessed through the estimation of haplotype diversity (Hd), and nucleotide diversity (π) in ARLEQUIN 3.1 (Excoffier et al. 2005).

To test if there is evidence of genetic structure among the four geographic groups, we performed a hierarchical analysis of molecular variance (AMOVA) using pairwise differences. In addition, to test if phenotypic divergence is related to genetic structure we also performed an AMOVA between gray-plumaged subspecies and yellow-plumaged subspecies. Genetic divergence between groups was also measured using *F_ST_* fixation index values (Wright 1931, 1978), which were interpreted following the guidelines in Hartl and Clark (1997). All tests were performed with ARLEQUIN 3.1 (Excoffier et al. 2005); and their significance was assessed using 1000 permutations.

To test for evidence of recent demographic changes in *A.albinucha*, we estimated demographic dynamics experienced by the whole taxon through the calculation of neutrality tests corresponding to Fu's F_S_ statistic (Fu 1997) and Tajima's D statistic (Tajima 1989). Significance of these tests (p < 0.02 in the case of the F_S_ statistic) was calculated by developing 1000 simulations using ARLEQUIN 3.1. Evidence of historical signatures of fluctuations in population size was also examined through a Bayesian skyline plot model on the Maximum Clade Credibility Tree (MCCT), as implemented in BEAST v1.8.4 (Drummond et al. 2012), using a coalescent-based estimation of population size changes over time with MCMC sampling procedure (Ho and Shapiro 2011, Houston et al. 2014).

### Analysis of the historic range using ecological niche models

We tested the hypotheses that the ecological/environmental conditions in which *A.albinucha* ranges at present may have allowed for population connectivity at least since the Last Interglacial (120,000 ya) using ecological niche models (ENM). We compiled a total of 475 geographical records, representing 176 localities, of the species through the Global Biodiversity Information Facility (GBIF, http://www.gbif.org) and museum vouchers (see Acknowledgements). GBIF records were filtered for elimination of both duplicates and records lacking geographic data. ENMs were obtained using 19 bioclimatic variables with a cell resolution of 2.5 arc-minutes (ca. 4.5 km^2^) generated by the Community Climate System Model (CCSM) downloaded through WorldClim (http://www.worldclim.org/bioclim; Hijmans et al. 2005). ENM models were obtained and evaluated in MAXENT 3.3.3 (Phillips et al. 2006), whose algorithms have been used to transfer present niche space conditions into past scenarios (120,000 ya–present). Past ENM reconstructions were based on the CCSM scenarios for the LIG (ca. 120,000 ya), Mid-Holocene (MHCO, ca. 6,000 ya), and LGM (ca. 22,000 ya). CCSM scenarios were preferred over other models as it has been proposed that global cooling conditions are not overestimated (Fernández-Mazuecos and Vargas 2013, Harrison et al. 2014), therefore representing a conservative hypotheses of humid montane forests connectivity. MAXENT parameters were run as follows: Ten-thousand random points within extreme coordinates 22N–105W, 20S–62W were generated to serve as background data to encompass mostly montane habitats in the Neotropics, 50 bootstrap replicates with a maximum iteration value of 5000, and a random test percentage of 25 with a 10 percentile training presence threshold rule (Warren and Seifert 2011, Baldwin 2009). To evaluate the predictive ability of the generated distribution models we implemented two model validations using the ROC plot method. As a first evaluation measure, we used the value for the area under the receiver operating characteristic curve (AUC), which can be used as a measure of the model's overall performance; for a second model validation we used PARTIAL-ROC (P-ROC) analysis (Barve 2008, Peterson et al. 2008), which generates ratios that provide a measurement of the correct identification of presences against the total area predicted (Mendoza-González et al. 2016). ENM models were visualized in ARCMAP 10 (ESRI 2010).

## Results

### Phylogeography

**Figure 2.**
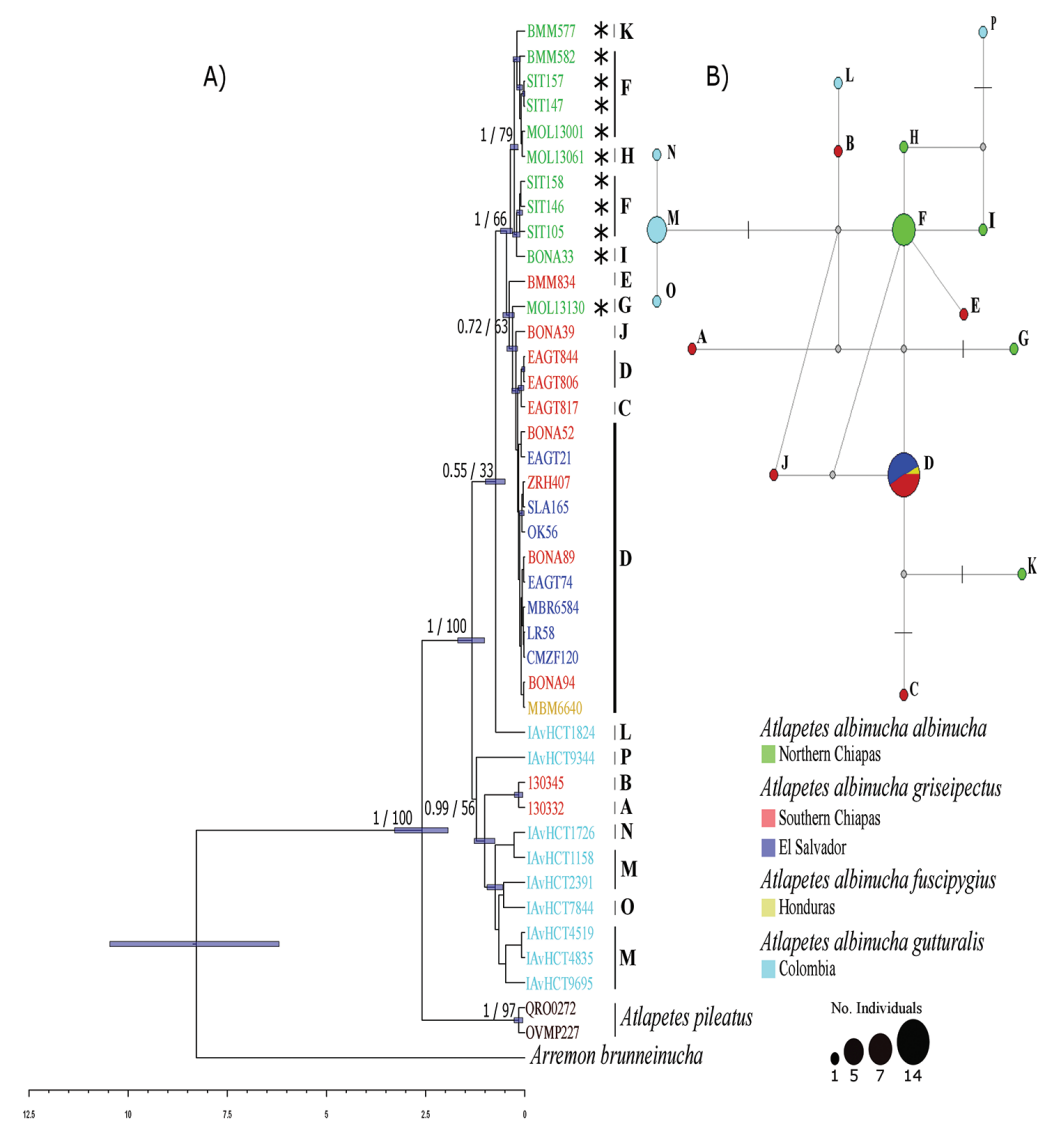
**A** Dated Bayesian maximum clade credibility tree showing phylogenetic relationships among members of *Atlapetesalbinucha* species. Node bars depict 95% HDP interval, scale bar represents millions of years. Nodal values above branches indicate posterior probabilities/ bootstrap supports of BI/ML. Capital letters depict haplotypes. An asterisk (*) indicate birds representing yellow morphs **B** Median-joining haplotype network for the concatenated dataset. Each color depicts the geographic provenance of samples: green-northern Chiapas (subspecies albinucha), red-southern Chiapas (subspeciesgriseipectus), blue-El Salvador (subspecies griseipectus), yellow-Honduras (subspecies fuscipygius) and light blue-Colombia (subspecies gutturalis). Each branch represents a single nucleotide change, transversal black lines along branches depict the occurrence of three mutations. Gray dots indicate median vectors inferred for the data.

Phylogenetic reconstruction analyses were conducted using the substitution models HKY+I+G for the first position of ND2 and the second position of Cyt b, F81+I for ND2 second position and third Cyt b position and HKY+G for the third and first positions of ND2 and Cyt b respectively. Both phylogenetic reconstruction methods (ML and IB) rendered similar topologies. The Bayesian tree topology (Figure 2A) recovered all of the *A.albinucha* samples in a monophyletic group (as suggested previously by Sánchez-González et al. 2015) showing two major well-supported clades (0.99 and 1.00) but moderate bootstrap support (0.56 and 0.66). One clade, including only gray-plumaged birds, grouped all of the individuals from the Central Andes of Colombia, as well as two individuals from southern Chiapas, in Mexico (Reserva Ecológica El Triunfo). The other clade included both yellow- and gray-plumaged birds from Mexico and Central American, as well as one Colombian sample from Serranía de los Yariguies, Santander (IAvH-CT 18248). No one of the four subspecies included was monophyletic according to the two mtDNA loci analyzed, and the yellow- and gray-plumaged groups were neither reciprocally monophyletic. Therefore, no clear phylogeographic structure was recovered from our analysis (Figure 2).

### Population genetic structure and demographic history

Overall, genetic diversity values for the geographic groups showed a low nucleotide diversity (π < 0.008), but high haplotype diversity (Hd > 0.71; Table 2). The AMOVA between geographic groups indicated that genetic variation was explained (almost equally) by both differences among populations (50.7%), as well as by variation within groups (49.2%, Table 3). Comparisons among pairs of geographic groups using *F_ST_* values revealed very high genetic differentiation between El Salvador and northern Chiapas (*F_ST_* = 0.71412), but lower differentiation was among El Salvador and southern Chiapas (*F_ST_* = 0.04556); the remaining *F_ST_* values indicated moderate genetic differentiation (*F_ST_* between 0.4 and 0.7). AMOVA between gray- and yellow-plumaged morphs showed that most of the variance occurs within groups (64.5%), paralleling the results of the phylogenetic analysis that showed that both phenotypes are not reciprocally monophyletic.

**Table 2.** Genetic diversity measures and demographic fluctuation measured at the species and population level within the concatenated data set. Abbreviations: N Sample size, Hd Haplotype diversity, π nucleotide diversity, SD standard deviation.

	**N**	**Hd (SD)**	**π (SD)**	**Tajima's D**	**Fu's F_s_**
**Northern Chiapas**	11	0.982 (0.046)	0.00264 (0.0015)	-1.49107	-2.654
**Southern Chiapas**	11	0.727 (0.144)	0.007674 (0.0041)	-1.037	-0.905
**El Salvador**	7	0.714 (0.181)	0.00077 (0.00028)	-1.023	-0.538
**Colombia**	9	0.722 (0.159)	0.004313 (0.0026)	-1.37093	0.81161
**Honduras**	1	–	–	–	–
**Total**	39	0.835 (0.047)	0.00652 (0.0007)	-1.267	-5.08

**Table 3.** Analyses of molecular variance (AMOVA) between gray- and yellow-plumaged morphs and between geographical groups.

**AMOVA: gray- and yellow-plumaged morphs**				
Source of variation	**Sum of squares**	**Variance components**	**Percentage of variation**	***P***
**Among morphs**	70.621	4.00944	35.48	0.001
**Within morphs**	269.815	7.29230	64.52	0.00098
**AMOVA: geographic groups**				
Source of variation	**Sum of squares**	**Variance components**	**Percentage of variation**	***P***
**Among geographic groups**	170.961	5.13057	50.72	0.001
**Within geographic groups**	169.475	4.98455	49.28	0.001

The haplotype network showed three non-shared high frequency haplotypes: one including most samples from Colombia (*gutturalis*); a second one where most samples from northern Chiapas (*albinucha*) are located; and a third high frequency haplotype that was shared by most samples corresponding to northern Central America, which includes subspecies griseipectus and *fuscipygius* (Figure 2B).

Despite relatively low bootstrap values, molecular dating of the divergence between *A.albinucha* and *A.pileatus* yielded a time estimate during the Late Pliocene-Early Pleistocene about 2.5 Mya (HPD range 1.94–3.28 Mya), whereas differentiation between major clades of *A.albinucha* apparently occurred around 1.5 Mya (HDP range 1.01–1.61 Mya), during the Early Pleistocene (Figure 2A).

**Figure 3.**
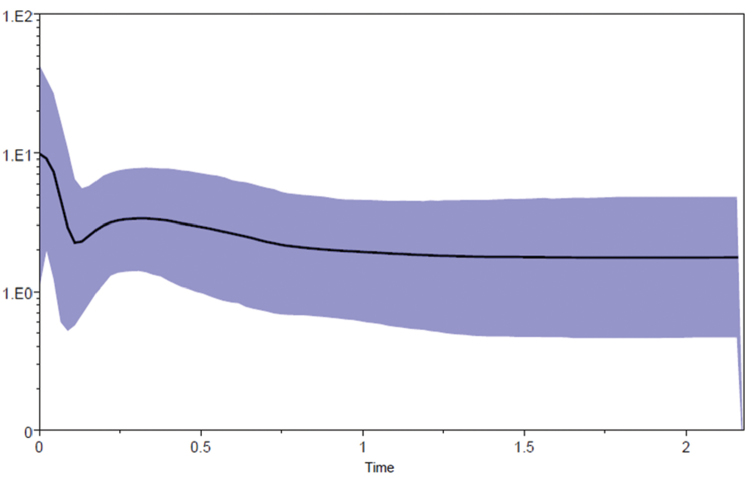
Bayesian skyline plot derived from the concatenated gene dataset of *Atlapetesalbinucha* species. Time in millions of years. Population size change (Ne*generation time) in the Y axis. Mean estimate is shown as a thick solid line, and the 95% HDP limits are shown in solid purple color area surrounding the mean estimate.

Historical demography in *A.albinucha* as estimated from the Tajima's D and Fu's F_S_ tests showed in most cases negative values, except for the F_S_ in the Colombian population. Both demographic tests did not depart from neutrality given that values were not significant neither at the species nor at the geographic group level; therefore, demographic fluctuations are difficult to suggest based on these values (Table 2). Bayesian skyline plot indicated an overall pattern of population stability throughout the Pleistocene at the species level, however, a fluctuation near the present (ca. 100,000 ya), suggests a population bottleneck followed by rapid population expansion (Figure 3), thus paralleling results from the genetic diversity analyses.

### Ecological niche models

**Figure 4.**
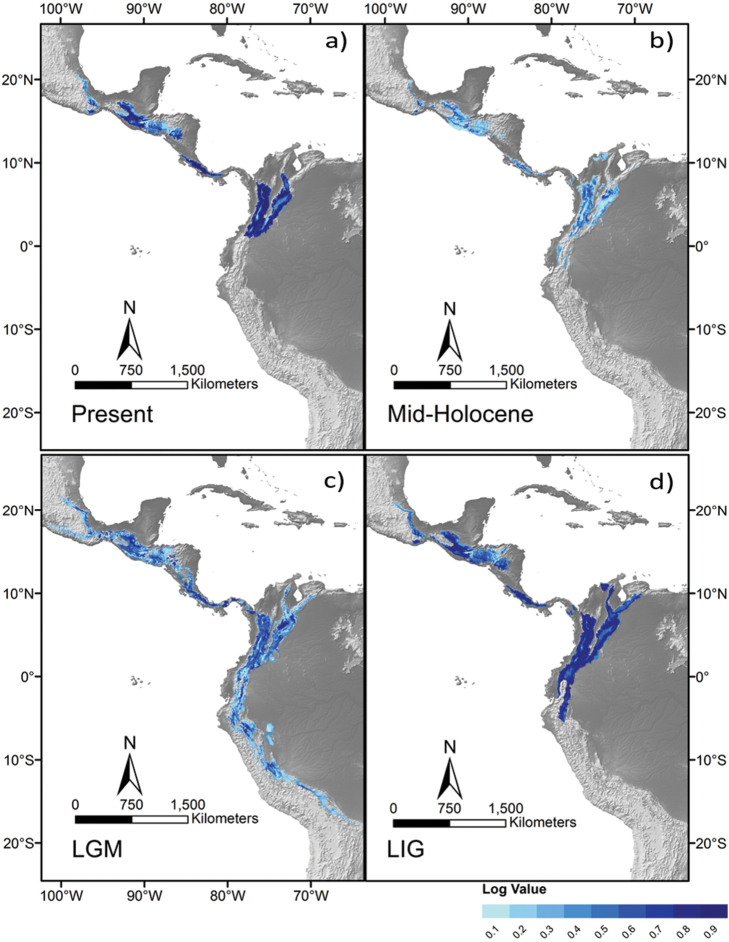
Maxent ENMs for *A.albinucha* species projected into present and past scenarios. Darker blue areas depict higher logistic prediction values. ENM projected in the **a** present **b** the Mid-Holocene Climatic Optimum (MH) **c** the Last Glacial Maximum (LGM) and **d** the Last Interglacial (LIG).

All of our ENM analyses performed better (AUC > 0.94) than a random non-predictive model (AUC = 0.5), indicating that the models obtained may reflect, relatively well, the past distribution of environmental conditions where *A.albinucha* inhabits at present. ENMs suggested a scenario of geographically fragmented environmental conditions for populations in Mexico, Central America, and Colombia during three of the modeled timeframes: LIG (ca. 120,000 ya, P-ROC, min = 0.998, max = 1.972; Figure 4d), Mid-Holocene (MH, ca. 6,000 ya, P-ROC, min = 0.997, max = 1.907; Figure 4b), and for the present (Figure 4a). Present, LIG, and MH timeframes showed four main environmentally suitable areas for *A.albinucha* separated by lowlands such as the Isthmus of Tehuantepec, the Nicaragua Depression, and the Isthmus of Panama. In contrast, ENM for the LGM (ca. 22,000 ya, P-ROC, min = 1.1404, max = 1.532) suggested these same lowland areas as corridors, which may have served for the dispersal between previously isolated populations (Figure 4c), thus supporting the scenario suggested by phylogeographic patterns.

## Discussion

The major result of our analyses using mtDNA sequence data for individuals of *A.albinucha* is that this taxon exhibits an incomplete genetic differentiation along their range in the Neotropical Montane Forest. The lack of clear phylogeographic structure in this montane bird taxon is in sharp contrast with expectations based on plumage differentiation which has resulted in the recognition of up to eight subspecies (Gill and Donsker 2018) as well as to the genetic divergence found in other birds from other naturally fragmented environments throughout the Neotropics (García-Moreno et al. 2004, Cadena et al. 2007, Navarro-Sigüenza et al. 2008, Weir et al. 2008, Pérez-Emán et al. 2010, Arbeláez-Cortés and Navarro-Sigüenza 2013). Despite not recovering a clear phylogeographic structure, the geographic distribution of the genetic variation in the geography is not completely random (as indicated by AMOVA and *F_ST_* values), suggesting a scenario in which two groups (South America and Mesoamerica) have been diverging in isolation followed by range expansion, allowing the mixture of the genetic variation in periods favoring habitat connectivity during the LIG. Signatures for this vicariant scenario of genetic differentiation may be found in the phylogeographic topology, in which two groups including mainly South American and mainly Mesoamerican individuals were recovered, and in the significant variation among geographic groups determined by the AMOVA analyses (Table 3), as well as in the gene flow values (Table 4). Moreover, the low nucleotide diversity, but high haplotype diversity we found for mtDNA of *A.albinucha*, is thought to be consistent with populations passing through genetic bottlenecks followed by rapid population growth (see Grant and Bowen 1998).

**Table 4.** Population pairwise comparisons using the concatenated data set. Above the diagonal is found the number of migrants per generation estimates (Nm value). Below the diagonal FST index. FST values with * depict significant values p < 0.05. Numbers depict geographic group correspondence.

	**1**	**2**	**3**	**4**	**5**
**1-Northern Chiapas**		0.70586	0.20016	0.2547	0.45921
**2-Southern Chiapas**	0.41464*		10.47426	0.67099	–
**3-El Salvador**	0.71412*	0.04556		0.24878	–
**4-Colombia**	0.66252*	0.42699*	0.66775*		0.75137
**5-Honduras**	0.52126	0	0	0.39956	

The phylogeographic pattern of *A.albinucha* is consistent with allotypy, a term used to denote a stage in intermediate polyphyly (Omland et al. 2006). Allotypy is a likely intermediate stage of divergence characterized by local fixation of haplotypes on the path to reciprocal monophyly (Hudson 1990). This genetic pattern has been found in other bird species such as ducks (Peters et al. 2005, Peters and Omland 2007) and ravens (Omland et al. 2006) in the Holarctic, a raptor species in Neotropical lowlands (Johnson et al. 2007), and in passerines from Australia (Joseph and Wilke 2007) and from the Neotropical montane forests (Arbeláez-Cortés et al. 2010). For most of these cases, present distribution of the genetic diversity may reflect the retention of the genetic diversity found in the ancestor for a long time after isolation, which may have had a larger population size, thus increasing the time for some polymorphisms to be retained (Joseph and Omland 2009). Similarly, population expansion derived from populations with high effective sizes may also explain the haplotype and nucleotide diversities observed (Harpending et al. 1998, Ray et al. 2003, Ng et al. 2013, Jezkova et al. 2015).

In the case of *A.albinucha*, BSP (Figure 4) suggests a long period of population stability, with a slight increase from 750,000 to 250,000 ya, after which a relatively slight decrease in population size occurred ca. 100,000 ya. This population decrease, followed by rapid population growth and range expansion is coincident with the Last Glacial Maximum during the Late Pleistocene (~21,000 ya), when colder conditions may have allowed the formation of corridors between previously isolated humid montane forest patches (Figure 4a), likely enhanced by the downward altitudinal range changes of the forest belt (Hooghiemstra et al. 2006, Gutiérrez-Rodríguez et al. 2011, Rojas-Soto et al. 2012, Ornelas et al. 2010, 2013, Ramírez-Barahona and Eguiarte 2013). Such a scenario, probably promoted gene flow between previously isolated populations (e. g., Hewitt 2000, Zink and Blackwell-Rago 2000, Weir 2006, Hooghiemstra et al. 2006, Barber and Klicka 2010, Pérez-Emán et al. 2010, Wachowiak et al. 2013, Bagley and Johnson 2014, Ornelas et al. 2016). In addition, an interesting result emerging from our ENM is that regions inhabited by yellow-plumaged and grey-plumaged populations in Chiapas-Guatemala have apparently never been isolated, which seems to support conclusions by Paynter (1972, 1978) regarding the weakness of a low river valley as an effective barrier in separating these populations.

Causes of differentiation in plumage coloration in *A.albinucha* remain elusive in our analysis, as both plumage coloration patterns appeared intermixed in the tree topology, which suggest different processes for the configuration of the genetic variation and the phenotypic plumage differentiation. Therefore, the clear phenotypic differentiation between yellow-colored birds in northern Chiapas and gray-colored birds in the rest of the distributional range suggests that plumage may be under natural selection. Similar results have been obtained for other groups of birds in different geographical and ecological settings, such as in the Tropical Pacific islands (Filardi and Smith 2005, Uy et al. 2009), shorebirds (Rheindt et al. 2011), and Australian woodswallows (Joseph et al. 2006). In the case of *Atlapetes* brushfinches, yellow- and gray-plumage patterns are apparently ecologically segregated from each other at different elevations. Gray-plumaged birds tend to occupy high elevation, whereas yellow-plumaged birds tend to occupy lower elevations (Remsen and Graves 1995), thus suggesting that gray-plumages have evolved to deal with conditions on high elevations, but also some dry low-elevation environments (Sánchez-González et al. 2015). Similar changes in plumage patterns like the one detected in our study, and their correlation with environmental variables, have been also documented for other bird groups throughout the World (see Bowers 1960, Hall et al. 1966, Wunderle 1981, Galeotti and Cesaris 1996, Grunst et al. 2014, Reudnik et al. 2015). At the molecular level, plumage color changes are apparently a result of the concentration of lutein in the feather (Johnson and Brush 1972, Brush and Johnson 1976, McGraw and Hill 2006), however the specific mechanism in *Atlapetes* is unknown, although some studies point to single point mutations at the MCR-1 (melanocortin-1 receptor gene) as responsible for similar plumage changes in birds (reviewed in Mundy 2005, but see Cadena et al. 2011). Consistent with previous molecular-based studies, this study also supported that mtDNA variation does not correspond to plumage pattern differentiation in this species (Sánchez-González et al. 2015), suggesting that plumage coloration pattern in *A.albinucha* may be taxonomically misleading because it doesn’t reflect population history. In addition, it has been shown that vocal repertories are very similar and calls between color morphs cannot be reliably differentiated (Sánchez-González et al. 2015, Boesman 2016).

Results in this paper are not conclusive in terms of the currently accepted taxonomy for *A.albinucha*. Genetic divergence as a result of allotypy is apparent, suggesting that these taxa are likely at allotypy (Omland et al. 2006). Results in other bird taxa where allotypy has been found, show support to maintain recognized species, as genetic divergence is accompanied by morphological divergence (e. g., Peters and Omland 2007, Johnson et al. 2007), whereas others advocate to a single widespread (albeit genetically differentiated) species (e. g., Peters et al. 2005, Omland et al. 2006), lending an ambiguous support for species recognition. The study of Johnson et al. (2007) offered however, a threshold for species and subspecies recognition for a Neotropical raptor. The application of such a threshold for *A.albinucha* would support a subspecific status for all populations analyzed, thus maintaining the current taxonomic treatment (Paynter 1978, AOU 1998, Gill and Donsker 2015). However, further studies should be extended to include southern Central American populations and other genetic markers. Finally, results presented here underline that a general pattern for the evolution of montane bird taxa in Mesoamerica and Northern South America should consider several exceptions like the one depicted here for *A.albinucha*, as well as emphasize the role of idiosyncratic events in the recent evolution of bird taxa in this region, as it has been suggested for lowland bird taxa (Smith et al. 2014).

## Conclusions

Genetic patterns found in *A.albinucha* were unexpected given previous findings in birds and other taxa codistributed in montane forests throughout the region (see Ornelas et al. 2013), which in general have showed corresponding patterns of genetic and morphological divergence (e. g., García-Moreno et al. 2004, Pérez-Emán et al. 2010).

The phylogeography of *A.albinucha* is consistent with allotypy, which has been suggested to represent an intermediate stage in the path to reciprocal monophyly (Omland et al. 2006). Most cases of allotypy have been reported in temperate birds from Eurasia (Peters et al. 2005, Omland et al. 2006, Peters and Omland 2007) as well as in the Eremian birds from Australia (Joseph and Wilke 2007), as well as in the Neotropics (see also Arbeláez-Cortés et al. 2010).

Environmental factors may have played a major role in shaping the evolution of morphological traits by natural selection that have been considered taxonomically relevant (Ball and Avise 1992), such as coloration pattern seen across the entire lineage (Paynter 1972, 1978, Remsen and Graves 1995, Sánchez-González et al. 2015), but that are not congruent with the genetic divergence indicated by mtDNA.
